# Measuring the effectiveness of an intensive IPV training program offered to Greek general practitioners and residents of general practice

**DOI:** 10.1186/1472-6920-13-46

**Published:** 2013-03-28

**Authors:** Maria Papadakaki, Eleni Petridou, Manolis Kogevinas, Christos Lionis

**Affiliations:** 1 Clinic of Social and Family Medicine, Faculty of Medicine, University of Crete, Voutes, P.C, Heraklion, 71003, Greece; 2 Department of Hygiene, Epidemiology and Medical Statistics, Athens University Medical School, 75 Micras Asias Ave, Goudi, Athens, 11527, Greece; 3 National School of Public Health, Alexandras Avenue 196, Athens, PC 115 21, Greece

**Keywords:** Intimate partner violence, General practitioners, Residents, Primary health care, Training, Knowledge

## Abstract

**Background:**

The need for effective training of primary care physicians in the prevention, detection and handling of intimate partner violence (IPV) has been widely acknowledged, given its frequency in daily practice. The current intervention study aimed to measure changes in the actual IPV knowledge, perceived knowledge, perceived preparedness and detection ability of practicing general practitioners (GPs) and general practice residents, following an intensive IPV training program.

**Methods:**

A pre/post-test design with a control group was employed to compare changes in baseline measures of IPV at the post intervention stage and at 12 months. A total of 40 participants provided full data; 25 GPs (11 in the intervention and 14 in the control) and 15 residents (intervention only). Three scales of the PREMIS survey were used to draw information on the study outcomes.

**Results:**

The training program met high acceptance by both groups of participants and high practicality in clinical practice. The GPs in the intervention group performed better than the GPs in the control group on “*Perceived preparedness*” and “*Perceived knowledge*” in both the post-intervention (*p = .012, r = .50 and p = .001, r = .68*) and the 12-month follow-up (*p = .024, r = .45 and p = .007, r = .54*) as well as better than the residents in “*Perceived preparedness*” at post-intervention level (*p = .037, r = .41*). Residents on the other hand, performed better than the GPs in the intervention group on “*Actual knowledge*” at the 12-month follow-up (*p = .012, r = .49*). No significant improvements or between group differences were found in terms of the self-reported detection of IPV cases.

**Conclusion:**

Further studies are needed to decide whether residency training could serve as an early intervention stage for IPV training.

## Background

Primary care is an important early intervention site for intimate partner violence (IPV), because general practitioners (GPs) have an ongoing therapeutic relationship with the whole family [1-3]. GPs are thought to come across large numbers of abused patients and it has been noted that a GP in full time practice is likely to see on a weekly basis, up to five women who have experienced abuse in the past year [4,5]. Despite the high prevalence of the problem in primary care settings, GPs will often say that they do not see many patients who have suffered violence [5]. This low physicians’ awareness has been investigated thoroughly in past research [6] and what always comes up as a conclusion is the need to inform future educational interventions and progress medical education in order to manage an improved physicians’ response to IPV [7-10]. Despite this call for IPV education, training interventions aiming to improve GPs’ response are rare [2,3,11,12] and although their results have been somehow encouraging, their overall effectiveness remains uncertain [12,13].

Medical education on the other hand is striving to find its way within the emerging demands for IPV training. Medical schools have started expanding their curriculums to include IPV education, but this early training has been frequently shown to be inadequate to address the problem and rarely reinforced during residency and continuing education [14-16] and most importantly, evidence on its efficacy on the subsequent behaviours and attitudes of trainees is scarce [17]. The situation is no better in general practice residencies, which have been critisised for inadequately preparing residents to address IPV [7,18]. As a result, residents have been shown in several studies to lack awareness and preparedness to deal with IPV in everyday practice [7,15,16] or be less effective than residents and physicians of other specialties in managing IPV [19].

As residency training is thought to serve as an early and well-timed intervention stage for IPV training [7,20], the need to optimize residency training on IPV has been emphasized [21,22]. However, incorporating family violence education into the standard residency curriculum is still thought to be challenging, considering that the overall learning requirements of residency programs continue to increase [23]. Interestingly, it has been suggested that educational efforts to increase IPV knowledge and skills of general practice residents need to utilise an intensive training program that is designed to meet certain training needs and takes into account residents’ time constraints [23]. In this heavy training schedule, evidence on the effectiveness of IPV training at this stage of medical education needs to be strengthened in order for IPV education to gain attention and recognition among the other learning requirements within residency programs. In fact, educational efforts to prepare a new generation of knowledgeable GPs through increasing IPV knowledge and skills of general practice residents need to underline why IPV education is more appropriate during residency training than during other stages of the medical education or professional life.

Nevertheless, international literature seems to lack educational efforts on IPV targeting both residents and practicing physicians of the same specialty and thus it is difficult to reach a conclusion on the period that continuous medical education on IPV could ensure better educational outcomes. Moreover, current educational approaches need reassessment as many have not shown sustained changes in attitudes and actual rates of IPV screening in clinical practice [24-26]. The numerous educational interventions of mixed success [8,11,27-30], indicate that we still lack the knowledge on how to train GPs in this subject and several questions such as “what works better”, “when is better to train GPs on this subject?” remain unanswered [12,31,32]. Under these circumstances, implementing a successful IPV training program within the realities of today’s medical world remains a significant challenge [33].

In Greece, the challenge is even greater as there are no opportunities of undergraduate, postgraduate or continuous education on IPV for health care providers. This educational gap is intensified due to the lack of professional guidance on the identification and management of IPV (e.g. clinical protocols and guidelines) and the minimal referral resources (6 shelters, 2 consultation centres). Despite recent efforts of the Greek government to increase the number and the capacity of referral resources, the available services are still centralized in major cities and operate under limited financial resources. Most importantly, there is no national agency designated to operate or supervise these services, resulting in dissimilar recording, documentation and intervention practices. The family violence law (L3500/2006) has been the most promising initiative in the prevention and management of family violence of the past decade, which failed however to integrate the health care sector among its provisions. Under these circumstances, increasing the knowledge, the skills and the capacity of Greek physicians on IPV identification and management, remains a pressing gap.

The current study was part of a broader research project carried out within the region of Crete in Greece with the overall aim to improve GPs’ response to IPV cases in primary health care. The necessity to improve health care response to IPV in Greece has been well justified in previous research [34-36]. This study reports on the evaluation of an intensive IPV training program offered to practicing GPs and residents of general practice. More precisely, the purpose of the study was three-fold: (1) to measure changes in the actual knowledge, the perceived knowledge, the perceived preparedness and the detection of IPV cases in the practicing GPs and the residents of general practice completing the training program, (2) to compare the changes in the aforementioned measures between the GPs and the residents of general practice completing the training program as well as between the intervention and the control groups, (3) to report on the logistics, the acceptability and the practicality of the training program. More precisely, we hypothesised that there would be significant changes in baseline measures of IPV (*a. IPV actual knowledge, b. perceived knowledge, c. perceived preparation, d. detection of IPV cases*) at post-intervention level and at 12 months follow up, in both groups of physicians completing the IPV training program. We further hypothesized that the GPs [Intervention group I] would present better study outcomes compared with the GPs not assigned to take the program (Control group) as well as better study outcomes compared with the residents of general practice [Intervention group II] due to differences in clinical experience. Finally, the intensive IPV training program was expected to meet high acceptability and practicality in both groups of physicians as its content drew on physicians’ training needs identified earlier through qualitative methods [37].

## Methods

### Setting and type of the study

A two-day IPV training program was offered to physicians serving primary care settings in the prefecture of Heraklion, one of the four prefectures of the Cretan region in Greece. Two different groups of physicians were targeted; a) practicing GPs serving rural health centres, and b) residents of general practice. A pre/post test design was employed to compare changes in baseline measures of IPV knowledge, attitudes and practice at post intervention stage and at 12 months. A control group was employed to compare changes in IPV measures between those assigned and those not assigned to take the program.

### Ethical approval

The project received a grant by the Research Committee of the University of Crete (Ref. No 263/05-10-2007) and written approval by two governmental bioethical bodies, operating at local and national level; the Bioethical Committee of the University Hospital in Heraklion, Crete (Ref. No 953/13-02-2009) and the National Centre for Health Research, EKEPI (Ref. No 6455/10-06-2009). The program was carried out in August 2010 at the prefecture of Heraklion in Greece and follow-up data were collected in August 2011. The local health authorities and the directors of the participating health units were officially notified about the training program and provided approval on the recruitment process and the methodology of implementation.

### Participants

All GPs (n = 78) serving rural health centres in the prefecture of Heraklion and all residents of general practice (n = 35) in the same geographically defined area were invited by the principal researcher to participate in the training program through face-to-face or telephone contacts. A list of names and contact details of the aforementioned groups of physicians, serving the area of interest, was obtained from the Regional Health Administration for the needs of the recruitment process, upon an official request. Physicians received a written invitation and material on the study objectives and expected outcomes. All physicians who accepted the invitation were conveniently assigned to an intervention or a control group, based on their availability during the period of the training. Physicians who reported inability to attend the training program due to personal or professional reasons, beyond their control, were assigned to the control group. Both groups were administered the same pre and post-intervention questionnaires concurrently. The two pre-intervention tests were used to assess the extent to which the two groups of providers were similar. Then the two post-intervention tests (intervention vs control group) were compared expecting to find a greater post-intervention measurement in the intervention group than the control group due to the effect of the intervention package. More than one physician per health centre could participate in the study and all the physicians serving a health centre were purposively assigned to the same study or control group. No financial or other incentives were foreseen for participation in the study. Participants who attended part of the training program or did not provide full data at post intervention stage or at 12 months follow up were excluded from the study.

### The training program

#### Logistics

The training program was implemented on 7–8 July 2010 at a University amphitheatre. Two afternoon sessions with a total duration of 9 hours, were necessary to cover all topics. Afternoon sessions were more convenient as they did not overlap with regular work duties of the participants. Early notification was arranged through advertising the event on the university website and the public media. Audiovisual equipment was arranged according to the educators’ needs. Handouts and other written material was reproduced and distributed to all the participants. Didactic and interactive learning methods were employed to meet the learning objectives. In didactic education the participants did not interact with the educators while in interactive education they participated actively in the teaching sessions.

### Training content

A panel of experts collaborated to develop the content of the IPV training program (Appendix I). Findings from a qualitative survey with Greek GPs were also taken into account in the design of the training content [37]. A number of barriers in responding effectively to IPV victims were identified in this study and were addressed through the training program. Major barriers identified by the GPs were the uncertainty regarding their role in the management of the victimized patients, the lack of confidence in their ability to accurately diagnose the problem, the discomfort when required to discuss IPV with their patients, the mistrust in the available referral services and the serious privacy and confidentiality issues affecting their recording practices. With respect to these findings, the training program included information and skill-building exercises designed to improve competencies in the identification, assessment, and documentation of abuse. Besides learning about the dynamics of IPV, participants developed skills in screening, interviewing, sensitive questioning, risk assessment, record keeping, and networking with local referral resources. It was further judged as important to provide participants with support for sustaining learning and the subsequent integration into practice through a range of additional sources, including specifically relevant policies, procedures and guidance, websites and literature from the field. They further had the opportunity to become acquainted with the “Shelter of abused women and children”, one of the few IPV referral resources operating at regional level. Three representatives of the NGO were invited to present the organization, its scope and activities using audio-visual material. Five more trainers, with a major scientific or professional involvement in IPV issues as well as representing different academic disciplines (GP, nurse, psychologist, social worker, and lawyer), were invited to deliver different parts of the training program. Two of the trainers were invited from other European countries purposively to share best practices in risk assessment and record keeping (UK, Belgium).

### Outcome measures

The Physician Readiness to Manage Intimate partner violence Survey (PREMIS, Appendix II) [38] was used to compare study and control groups. The PREMIS survey is a comprehensive and reliable self-administered measure of physician preparedness to manage IPV in four broad areas: (1) IPV background; (2) actual knowledge; (3) opinions; and (4) practice issues (self-reported management behaviours). The questionnaire has already been translated and validated in the Greek context by the authors of the current paper [39]. For the needs of the current study, 3 out of the 10 scales of the tool were employed, assessing participants’ actual IPV knowledge (10 items), perceived IPV knowledge (10 items) and perceived preparation to manage IPV at their clinical setting (14 items). Actual knowledge was assessed via a range of multiple choice questions (e.g. *what is the strongest single risk factor for becoming a victim of intimate partner violence, which of the following are warning signs that a patient may have been abused by his/her partner?)* and true/false questions evaluating type of risk and effects (e.g. *there are good reasons for not leaving an abusive relationship, when asking patients about IPV physicians should use the words abused or battered*). A summative score was calculated by allocating “1” for a correct response and “0” for an incorrect response. Perceived knowledge was examined through a number of questions answered on 7-point likert scales anchoring from 1 = nothing to 7 = very much (e.g. *how much do you feel you now know about signs or symptoms of IPV*, *how much do you feel you now know about what to say and not say in IPV situations with a patient)*. The perceived preparation comprised a number of items answered on 7-point likert scales anchoring from 1 = not prepared to 7 = quite well prepared (e.g. *how prepared do you feel to ask appropriate questions about IPV, how prepared do you feel to document IPV history and physical examination findings in patient’s chart).* Detection of IPV was assessed at baseline and at 12 months using a single open question adopted from the PREMIS survey with slight changes as follows: “*How many new diagnoses (picked up an acute case, uncovered ongoing abuse, or had a patient disclose a past history) of IPV would you estimate you have made in the past year*?”. Five response options were available as follows: 0 = none, 1 = 1-5, 2 = 6-10, 3 = 11-20, 4 = 21 or more. Information on the physicians’ prior training in IPV and other descriptive data were also collected. Participants completed the study questionnaire at three points during the study; a) before the training program, b) immediately after the training, and c) 12 months after the training.

Apart from the aforementioned questionnaire, participants were asked certain yes/no questions regarding the acceptability (“*did you find the training interesting?*"), and the practicality of the IPV training program (“*was this training of benefit to you?”*), as well as two open-ended questions regarding their suggestions for improvement and future action *(“what aspect of intimate partner violence would you have liked to have more information on during this training program?”, “what would help you to better address the problem at your practice?”*).

### Data analysis

Analysis was performed using SPSS 19.0. Descriptive statistics were used to describe physician profiles. For the needs of the analysis, the categorical variable that measured the new diagnoses in the past year, was transformed into a dichotomous variable with 0 (no new cases detected) and 1 (new cases detected). Chi-square tests and student *t*-test were employed to test for baseline differences in demographic items. The magnitude of the intervention’s effect was assessed based on the statistical significance of the effects as well as based on estimates of effect size. An effect size of .10 was considered as “small”, .30 was considered as “medium” and .50 was considered as “large”, based on Cohen’s proposed rules of thumb for interpreting effect sizes [40,41]. The non-parametric Mann–Whitney Test was used to detect differences in continuous variables between the two intervention groups (I and II) as well as between the intervention and the control groups (GPs). Chi-square test with a Monte Carlo simulation was applied to test differences in dichotomous data between the intervention groups (I and II) and between the intervention and control groups (GPs) [42]. Repeated measure, Wilcoxon signed rank test statistic was applied to test differences in the ordinal data at pre- and post-intervention level as well as at post-intervention and follow-up level, in each individual group of participants. An alpha value < 0.05 was selected. McNemar Test was used to examine changes in the dichotomous data at pre- and follow-up level, in each individual group of participants.

## Results

### Participant characteristics and flow through the study

**Figure 1:**
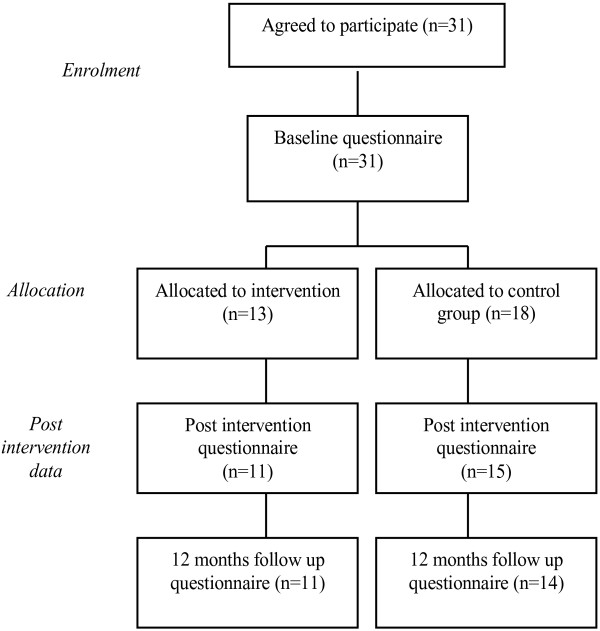
Flow diagram of progress through study (Practicing GPs).

**Figure 2:**
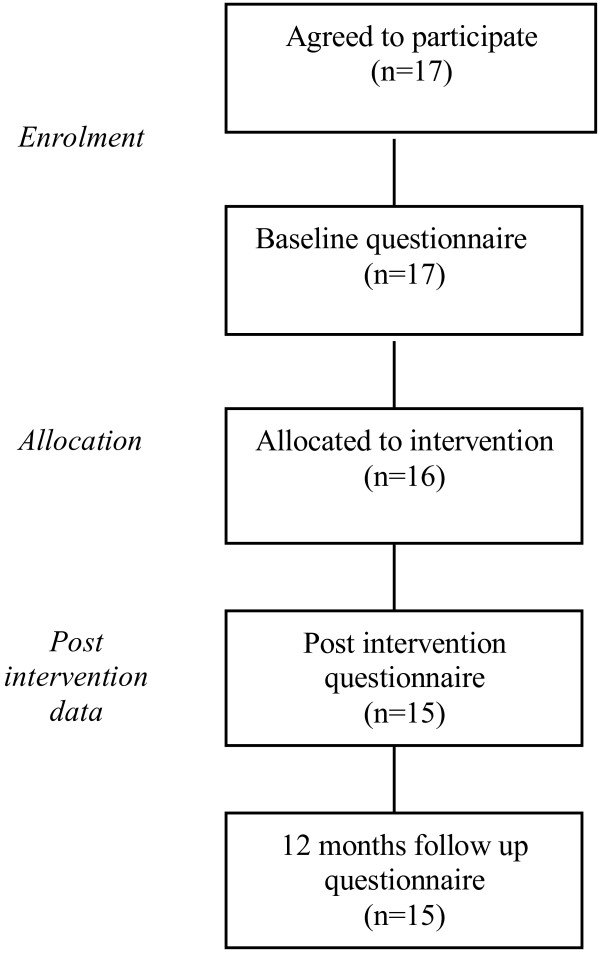
Flow diagram of progress through study (Residents of general practice).

As depicted in Figures 1 and 2, a total of 48 physicians accepted the invitation; 31 GPs (39.7% of all the GPs invited) and 17 residents of general practice (48.5% of all the residents invited). Out of the 31 GPs who agreed to participate, 13 were allocated to the intervention group [Intervention Group I] and 18 were allocated to the control group. After the training program had been concluded, 11 GPs had been retained in the intervention group and 15 GPs in the control group to provide post-intervention data. Twelve months after the training program, 11 GPs in the intervention group and 14 GPs in the control group provided follow-up data. All the residents of general practice who accepted to participate in the study were allocated to the intervention group [Intervention Group II] due to the limited number of candidates. A total of 15 residents provided post-intervention and 12-month follow up data.

As regards to the participants’ characteristics, detailed information is shown in Table 1. The majority of the GPs in both the intervention and the control group were women (63.6% and 78.6% respectively), while most of the residents of general practice were men (66.7%). Mean age and clinical experience were similar in the two groups of GPs, while residents were younger in age and without clinical experience. Previous IPV training was reported by less than 10% of participants in all the study groups of GPs and residents.

**Table 1:** Participants’ characteristics

	***GPs***	***GPs***	***Residents***
	***Intervention (n = 11)***	***Control (n = 14)***	***Intervention (n = 15)***
**Gender**	***n (%)***	***n (%)***	***n (%)***
*Men*	4 (36.4)	3 (21.4)	10 (66.7)
*Women*	7 (63.6)	11 (78.6)	5 (33.3)
Age*	39.6 (2.7)	40.8 (3.0)	36.0 (4.6)
Years in practice*	6.0 (2.0)	6.9 (2.9)	-
**Prior IPV training**	1 (9.0)	1 (7.0)	0 (0)

*Mean (Standard Deviation).

### Comparison between the intervention groups of GPs and residents of general practice in the preparedness, knowledge and detection of new cases

Detailed information on the differences between the GPs and residents of general practice in terms of their performance on the study measures are shown in Table 2. The analysis showed a significant effect of group in “*Perceived preparedness*”, with the GPs scoring higher than the residents at post-intervention level (*U = 42.5, p = .037, r = .41*). A significant effect of group was also found in “*Actual knowledge*” with the residents scoring higher than the GPs at the 12 month follow up (*U = 68.5, p = .46, r = .14*) (*U = 34.5, p = .012, r = .49*). Likewise, the number of residents who reported detecting new cases at 12 month follow-up turned out to be higher than the one of GPs, without this difference being statistically significant.

**Table 2:** Comparisons between the intervention group I (GPs) and the intervention group II (Residents) in perceived preparedness, perceived knowledge, actual knowledge and detection of new cases

	***GPs (n = 11)***	***Residents (n = 15)***	***Mann–Whitney Test***		
**Perceived Preparedness**	***Median (Min/max)***	***Median (Min/max)***	***U***	***Effect size (r)***	***P-value***
*Baseline*	3.55 (2.22-6.11)	3.00 (2.00-4.89)	54.0	.29	.138
*Post-intervention*	5.22 (3.67-5.89)	4.33 (2.67-6.00)	42.5	.41	**.037**
*12-month*	4.77 (3.67-5.89)	4.44 (2.56-6.00)	61.5	.21	.274
**Perceived knowledge**					
*Baseline*	3.06 (1.50-5.75)	2.93 (1.81-4.81)	71.5	.11	.567
*Post-intervention*	5.31 (3.38-5.94)	4.75 (2.00-6.00)	51.0	.32	.101
*12-month*	5.31 (3.38-5.88)	4.18 (2.38-6.00)	47.0	.36	.064
**Actual knowledge**					
*Baseline*	20.0 (16.0-27.0)	21.0 (13.0-27.0)	78.5	.04	.833
*Post-intervention*	27.0 (13.0-31.0)	26.0 (20.0-30.0)	68.5	.14	.464
*12-month*	21.0 (15.0-28.0)	25.0 (20.0-31.0)	34.5	.49	**.012**
**Detection of new cases**^**1,2**^	*n (%)*	*n (%)*	*χ2*	*df*	*p-value*
*Baseline*	5 (45.5)	6 (40.0)	.077	1	.781
*12-month*	7 (63.6)	11 (73.3)	.280	1	.683

^1^ n(%) of GPs/residents who detected new cases in the past year.

^2^ Chi-square test, Monte Carlo simulation, level of statistical significance .05.

### Comparison between the intervention and the control group of GPs in the preparedness, knowledge and detection of new cases

Differences between the intervention and the control group in terms of their performance on the study measures are shown in Table 3. A Mann–Whitney test indicated a significant effect of group in “*Perceived preparedness*” and “*Perceived knowledge*”. The intervention group of GPs scored higher than the control group in both measures at post-intervention (*U = 31.0, p = .012, r = .50 and U = 15.0, p = .001, r = .68 respectively*) and at 12 month follow-up (*U = 36.0, p = .024, r = .45 and U = 27.5, p = .007, r = .54 respectively*). Although the intervention group scored higher than the control group in “*Actual knowledge*” at both the post-intervention and the 12 month follow-up, a significant effect of group was only shown at the 12 month follow-up (*U = 41.0, p = .047, r = .40*). On the contrary, the number of participants who detected new cases was greater for the control group than for the intervention group in both the pre-intervention level and the 12 month follow-up, without this difference being statistically significant.

**Table 3:** Comparisons between the intervention and the control group in perceived preparedness, perceived knowledge, actual knowledge and detection of new cases

	***Intervention***	***Control***	***Mann–Whitney Test***		
	***(n = 11)***	***(n = 14)***			
	***Median (Min/max)***	***Median (Min/max)***	***U***	***Effect size (r)***	***P-value***
**Perceived Preparedness**					
*Baseline*	3.55 (2.22-6.11)	4.77 (2.44-5.67)	50.0	.30	.138
*Post-intervention*	5.22 (3.67-5.89)	4.27 (2.78-6.11)	31.0	.50	**.012**
*12-month*	4.77 (3.67-5.89)	3.77 (2.33-5.44)	36.0	.45	**.024**
**Perceived knowledge**					
*Baseline*	3.06 (1.50-5.75)	3.59 (1.38-4.44)	72.0	.05	.784
*Post-intervention*	5.31 (3.38-5.94)	3.87 (2.25-5.44)	15.0	.68	**.001**
*12-month*	5.31 (3.38-5.88)	3.50 (2.19-5.19)	27.5	.54	**.007**
**Actual knowledge**					
*Baseline*	20.0 (16.0-27.0)	17.0 (8.0-26.0)	28.0	.54	**.007**
*Post-intervention*	27.0 (13.0-31.0)	17.0 (12.0-28.0)	43.0	.37	.061
*12-month*	21.0 (15.0-28.0)	18.0 (12.0-25.0)	41.0	.40	**.047**
**Detection of new cases**^**1,2**^	*n (%)*	*n (%)*	*χ2*	*df*	*p-value*
*Baseline*	5 (45.5)	9 (64.3)	.887	1	.435
*12-month*	7 (63.6)	12 (85.7)	1.646	1	.350

^1^ n(%) of GPs who detected new cases in the past year.

^2^Chi-square test, Monte Carlo simulation, level of statistical significance .05.

### Changes in GPs’ and residents’ preparedness, knowledge and detection of new cases over the study period

Detailed information on the participants’ performance on the outcome measures over the study period are presented in Table 4. The Wilcoxon signed-ranks test indicated that the training program had a large effect in both the “*Perceived preparedness*” and the “*Perceived knowledge*” of the intervention group of GPs [Intervention Group I], at post-intervention level (*Z = 2.0, p = .041, r = .62 and Z = 2.8, p = .006, r = .84 respectively*) and at 12 months follow up (*Z = 2.3, p = .023, r = .69* and *Z = 2.2, p = .027, r = .67*, respectively). The “*Actual knowledge*” showed no significant improvement over the study period in either of the two groups of GPs while a large effect size was shown in the “*Perceived preparedness*” of the control group of GPs at 12 month follow-up *(Z = 2.7, p = .007, r = .72)*. The analysis further indicated that the training program had a large effect on the “*Perceived preparedness*” of the intervention group of residents [Intervention Group II] at post-intervention level (*Z = 2.5, p = .011, r = .66*), which was not evident at the 12 month follow-up. A large effect size was also shown in the residents’ “*Perceived knowledge*” and “*Actual knowledge*” at post-intervention level (*Z = 3.0, p = .003, r = .77* and *Z = 2.5, p = .010, r = .66*, respectively) as well as 12 months after the training program (*Z = 2.2, p = .028, r = .57* and *Z = 2.5, p = .011, r = .65*, respectively).

**Table 4:** Changes in perceived preparedness, perceived knowledge, actual knowledge and detection of new cases over the study period

	***GPs (n = 11)***				***GPs (n = 14)***				***Residents (n = 15)***			
	***(Intervention group I)***				***(Control group)***				***(Intervention group II)***			
	***Baseline/Post***		***Post/12 month***		***Baseline/Post***		***Post/12 month***		***Baseline/Post***		***Post/12 month***	
	***Z***	***p-value (r)***	***Z***	***p-value (r)***	***Z***	***p-value (r)***	***Z***	***p-value (r)***	***Z***	***p-value (r)***	***Z***	***p-value (r)***
^*1*^*Perceived preparedness*	−2.048	**.041**	−2.271	**.023**	-.668	.504	−2.687	**.007**	−2.546	**.011**	-.255	.798
		(.62)		(.69)		(.18)		(.72)		(.66)		(.06)
^*1*^*Perceived knowledge*	−2.771	**.006**	−2.214	**.027**	−1.665	.096	−1.425	.154	−2.986	**.003**	−2.197	**.028**
		(.84)		(.67)		(.44)		(.38)		(.77)		(.57)
^*1*^*Actual knowledge*	-.892	.373	-.990	.322	-.663	.507	-.160	.873	−2.565	**.010**	−2.530	**.011**
		(.27)		(.30)		(.18)		(.04)		(.66)		(.65)
^*2*^*Detection of new cases*	***Baseline/12 month***				***Baseline/12 month***				***Baseline/12 month***			
	.625				.375				.180			

^1^Wilcoxon Signed-ranks Test (r) = effect size.

^2^McNemar Test.

### Issues of acceptability, practicality and suggestions for an improved response

Of the 25 participants who attended the training program, 23 participants evaluated the program as interesting, while 2 participants were unsure. As regards to the practicality of the training program, 21 participants evaluated the program as beneficial and 4 participants were unsure or negative. Only 16 participants provided their suggestions for improvement and future action. Most of them would have liked to have additional hours of training on communication and interviewing techniques (n = 12), followed by guidance about when to suspect IPV (n = 11), as well as information on the legal protection when reporting suspected cases to the state authorities (n = 9). As regards to the suggestions provided by the 16 participants for a better management of IPV cases in primary care settings, the most common was the delivery of lifelong IPV training to primary care providers (n = 16), followed by the establishment of social services in primary care districts and the increase in the number of specialized social workers serving these districts (n = 11), the development of a website offering information on IPV and referral resources (n = 8), the development of a network of support services for IPV operating at primary care level (n = 7) and lastly the introduction of IPV screening tools (n = 5).

## Discussion

This paper shows that the intensive IPV training program tested in the current intervention study had overwhelming acceptance and was regarded as beneficial to those who participated. Despite the small number of participants in the intervention groups, the study indicates that the IPV training program was successful in increasing physicians’ IPV perceived knowledge and preparedness and that these changes could persist over at least 12 months. These findings, although not generalizable to the whole population of Greek physicians, seem important as generally the effect of training tends to diminish in time [31,43]. Most importantly, these positive outcomes occurred in the absence of other organizational changes and this makes the current training program even more successful. The major success of the study is that it achieved changes in the educational outcomes only in the groups of physicians who completed the IPV training program and not in the control group. This strengthens our belief that the changes in the outcome measures are largely attributed to the IPV training program and not to other external factors. This is further evident in the comparison drawn between the intervention and the control group of GPs, where significant differences in favour of the intervention group emerged for most of the outcome measures. Our results seem to be consistent with the findings of other studies, which demonstrate significant improvement in knowledge, self-assessed skills, and attitudes after training [11,27].

What stands out of the results is the participants’ performance on actual knowledge, which although substantially improved in both intervention groups immediately after the training program, nevertheless only the group of residents retained the knowledge gains at 12-month follow up while the actual knowledge of GPs dropped considerably. Although this difference may be an effect of the ongoing residency training, it may also imply that the GPs who completed their training years ago are less adaptive to new knowledge compared with the residents who are still in the educational process. This could further suggest that training programs aiming to increase the actual knowledge on IPV are more effective when implemented during residency training than later in the professional lifespan. Finally, this finding may suggest that a single training program may not be sufficient to manage sustainability of knowledge gains in the long term without additional changes in practice or repetition of training.

Interestingly, when comparing the GPs and the residents who participated in this training program in terms of their perceived knowledge and preparedness, significant differences between the two groups were only shown in the measurements of perceived preparedness. In particular, GPs were shown to have higher perceived preparedness following the training program compared with the residents, which difference did not remain significant one year later. Although GPs were expected to feel more prepared than residents due to their differences in the clinical experience, this may also suggest that the intervention achieved to temporarily differentiate self-perceived preparedness between the two groups or that the ongoing training of residents increased their self-preparedness and compensated the initial difference. We should however acknowledge that since residents tend to report that their activity and skill levels are higher than they actually are [7], their self-perceived preparedness ratings may have been overly favourable in this study. This could partly explain why the improvement of residents’ actual knowledge was temporary and did not remain significant one year after the training program.

Another noteworthy outcome is that the study did not succeed in bringing a statistically significant change in the number of participants who reported detection of new IPV cases one year after the training program. Intervention studies have been shown to present mixed results in terms of their effectiveness to increase detection of domestic violence in the medical clinic [8,27]. This is however an issue of outmost importance as it could indicate physicians’ difficulty to apply their knowledge gains and their self-preparedness into everyday practice. Although it is possible that the training program itself was insufficient in connecting knowledge with clinical practice, we should not overlook the fact that other barriers may have been involved, such as system’s unpreparedness or time constraints that need to be addressed in conjunction with professional knowledge and self-preparedness to manage an improved physicians’ response to IPV.

Interestingly, the control group of GPs who reported detection of new cases at follow up was substantially higher compared with the intervention group of GPs, despite not participating in the training program. This finding cannot be attributed to higher levels of IPV knowledge as the performance of the control group on actual knowledge was weaker than the intervention group. Therefore, this finding could either be interpreted by the greater perceived preparedness demonstrated by the control group at baseline or by their increased alertness during the study period due to their awareness of being “surveyed”, known as the “Hawthorne effect”.

### Strengths and weaknesses

Certain weaknesses of this study should be mentioned for future reference in research and intervention studies. First, the study participants derived from one prefecture of Greece and the findings could not be generalized to all the Greek GPs and residents of general practice. Second, participants were not randomized but were conveniently allocated to the study groups and thus it is possible that highly motivated people were included in the intervention groups introducing a bias. Third, as a result of the lack of randomization, there was a significant difference detected between the intervention and the control group of GPs in the baseline measurement of actual knowledge, which limits the comparability of this outcome measure between the two groups. Fourth, there was no control group for the residents of general practice and this makes it difficult to identify whether improvements in the intervention group of residents were random. It should be mentioned however that evidence on the residents’ IPV knowledge and practices is lacking and thus any new information could be beneficial in addressing their competence deficits in this area. Fifth, evidence other than self-reports (e.g. patient-related measures) would have been more efficient in exploring physicians’ practices. However electronic patient records are not yet available in the Greek Primary Health Care and this limits the available patient-related information. Sixth, we used self-reports to measure participants’ preparedness and it is unknown how the subjective assessment of self-reported preparedness relates to performance. Nevertheless, research on physicians’ behaviour indicates that confidence in one’s ability to make a change (e.g., self-efficacy) is related to the likelihood of doing so [38]. Seventh, there was a low participation in the study, which was expected due to the time constraints. Financial incentives would have been more effective in increasing participation rates although this aspect would have introduced another bias in the study.

Despite the aforementioned limitations, the current training program has employed a rigorous methodological design involving a control group of physicians, a pre/post intervention assessment and a 12-month follow up, which ensured a longstanding monitoring of participants’ performance following the training program. Other strengths of this study are related to the content of the training program, which was designed to address existing knowledge/practice gaps of the Greek GPs [37] and the use of validated research instruments in the assessment of the study outcomes [39]. Most importantly, this study is among the few published studies, if not the first, that draws comparisons between practicing physicians and residents of the same specialty, in terms of IPV educational outcomes.

## Conclusion

Our study clearly demonstrates the importance of violence prevention training in improving young practitioners’ response to IPV. Based on the outcomes, residency training could serve as a suitable opportunity for IPV training as it could facilitate longstanding knowledge gains for residents. However, the current study did not manage to generate evidence on residents’ improved performance following the training program. Future efforts should employ randomized control designs to verify the current outcomes and extend our educational goals. In light of the outcomes of the current training program, it would be interesting to expand future IPV interventions by including medical students along with residents and practicing GPs in order to draw more firm conclusions about the period which is most convenient and effective to intervene.

## Competing interests

The author’s declare that they no competing interest.

## Authors’ contributions

MP conceived the study, participated in its design, acquired and interpreted the data and drafted the manuscript. EP and MK were involved in the interpretation of data, helped in drafting the manuscript and revisited the manuscript critically for important intellectual content. CL conceived the study, participated in its design and coordination and helped to draft the manuscript. All authors read and approved the final manuscript.

## Authors’ information

MP is a lecturer at the Department of Social Work at the Technological Educational Institute of Crete. She obtained a first degree in Social Work (TEI of Crete), holds an MPH and she is currently a PhD candidate at the Medical School, University of Crete. She is the Greek representative in a European Primary Health Care Network on Family Violence and is the co-author in a number of scientific papers on family violence.

EP a board certified pediatrician and social medicine professional, is currently serving as a Professor of Preventive Medicine and Epidemiology in Athens University Medical School and Director of the Center for Research and Prevention of Injuries (CE.RE.PR.I.). She is a member of the International Society for Violence and Injury Prevention (ISVIP) and has collaborated with the Harvard Injury Control Research Center in the development, standardization and implementation of a screening tool and educational package for intimate partner violence in emergency departments and primary health care settings in several European member states. She has a wide experience in injury prevention and control; pediatric and perinatal epidemiology; childhood leukemia and lymphomas; cancer epidemiology; epidemiologic studies related to diet, tobacco, and alcohol risky behaviour; health services research and clinical epidemiology. She has published more than 200 research studies and has received a series of awards for her work.

MK graduated at the Medical School of Athens, Greece and did his PhD in Epidemiology at the University of London (1989). He worked at the International Agency for Research on Cancer (IARC/WHO), Lyon, from 1989–1994. Since then he is at the Municipal Institute of Medical Research (IMIM) Barcelona and is co-Director of the Centre for Research in Environmental Epidemiology (CREAL). He is currently also Professor at the National School of Public Health in Athenes, Greece. He served in several WHO and other expert committees. He has published more than 250 scientific papers in peer-reviewed journals.

CL is a Professor of General Practice and Primary Health Care at the School of Medicine, University of Crete, Greece and the Director of the Clinic of Social and Family Medicine. He was recently appointed as the chairman of the International Federation of Practice based Research Network (IFPCRN). In the past he was elected as the vice chairman of the European General Practice Research Network and has also served the Executive Board of the European Society of General Practice/Family Medicine (WONCA). He is involved in an editorial and advisory capacity with a number of international journals and has published more than 200 papers in international journals.

## Pre-publication history

The pre-publication history for this paper can be accessed here:

http://www.biomedcentral.com/1472-6920/13/46/prepub
